# Epigenetic Clocks Identify Harmful Epigenetic Aging Linked to Depression Severity and Cognitive Deficits in Major Depressive Disorder

**DOI:** 10.14336/AD.2025.0781

**Published:** 2026-08-18

**Authors:** Chao Liufu, Tao Pang, Haohao Zheng, Lei Yang, Li Yang, Xuebing Huang, Ying Qian, Suhua Chang

**Affiliations:** ^1^Peking University Sixth Hospital, Peking University Institute of Mental Health, NHC Key Laboratory of Mental Health (Peking University), National Clinical Research Center for Mental Disorders (Peking University Sixth Hospital), Beijing, China.; ^2^Beijing Key Laboratory for Big Data Innovative Application of Child and Adolescent Mental Disorders, Beijing, China.; ^3^Henan Collaborative Innovation Center of Prevention and Treatment of Mental Disorder, the Second Affiliated Hospital of Xinxiang Medical University, Henan, China

**Keywords:** MDD, epigenetic aging, cognition

## Abstract

Major depressive disorder (MDD) is a prevalent mental illness characterized by significant morbidity and mortality. Cognitive impairment is a common feature of MDD, closely related to the aging process. Epigenetic aging calculated using DNA methylation is an important marker of biological aging. This study aims to investigate the relationships among MDD, epigenetic aging, and cognitive function. We assessed age acceleration using epigenetic clocks based on DNA methylation data in discovery dataset with 39 MDD patients and 40 healthy controls, and then validated the results in one independent MDD cohort with 359 cases and 68 controls. The results revealed that patients with MDD exhibited significantly greater age acceleration as measured by the DamAge clock and elevated mortality risk as indicated by the Zhang clock. Notably, the age acceleration of DamAge was positively correlated with depressive symptom severity. Epigenome-wide association study of the age acceleration of DamAge identified 1,472 significant CpG sites. Enrichment analyses further revealed that these CpG sites are potentially involved in cytoskeletal mechanisms, signaling pathways, and inflammatory response. Cognitive assessments showed significant correlations between emotion recognition task performance and age acceleration from multiple epigenetic clocks, suggesting a link between accelerated epigenetic aging and cognitive impairment in MDD. Our results underscore the potential role of epigenetic aging in understanding the biological underpinnings of MDD and its associated cognitive dysfunctions.

## INTRODUCTION

Major depressive disorder (MDD) is a prevalent mental illness and the leading cause of disability worldwide [1]. Its prevalence varies across different populations, with a 12-month prevalence of 10.4% and a lifetime prevalence of 20.6% in the United States [2]. An increasing body of evidence suggests that MDD is a significant risk factor for mortality [3] and is associated with various aging-related phenotypes and diseases, including cardiovascular disease [4, 5], diabetes [6, 7], obesity [8], cognitive impairment [9], and dementia [10], among others. This suggests that MDD may be associated with accelerated aging.

There has been increasing attention on biological age, which differs from chronological age. Biological aging is a complex process involving interconnected changes across multiple biological pathways, ultimately resulting in the accumulation of damage in cells and tissues [11]. Accumulating evidence indicates that hallmarks of biological aging are altered in MDD [11], including cellular senescence (e.g., telomere shortening [12, 13]), dysregulated intercellular communication (e.g., senescence-associated secretory phenotype factors [14], inflammaging [15]), mitochondrial dysfunction (e.g., excessive reactive oxygen species production [16], mitochondrial DNA damage [17]), epigenetic alterations (e.g., DNA methylation [18]), and genomic instability (e.g., DNA damage accumulation [19]). These findings collectively suggest that MDD is associated with accelerated biological aging processes. The estimation of DNA methylation age, also referred to as the epigenetic clock, is regarded as one of the most promising indicators of biological aging [20].

In recent years, researchers have developed various epigenetic clocks to estimate biological aging. First-generation epigenetic clocks, such as the Horvath [21] and Hannum [22] clocks, were specifically designed to predict chronological age. Subsequently, 'second-generation' epigenetic clocks have been introduced, including PhenoAge [23], GrimAge [24], and Zhang [25] clocks. Unlike their predecessors, these advanced clocks are not solely focused on predicting chronological age; instead, they aim to enhance the accuracy of predictions related to aging and mortality outcomes. Recently, Ying et al. [26] have introduced a novel framework that integrates causal information into epigenetic clocks, resulting in the establishment of two distinct clocks: DamAge and AdaptAge. DamAge and AdaptAge are novel epigenetic clocks that represent a new frontier in the assessment of epigenetic aging [27]. DamAge is designed to monitor detrimental methylation changes and is associated with negative outcomes, including mortality, whereas AdaptAge tracks adaptive methylation changes and is associated with beneficial adaptations. While numerous epigenetic clocks [28-33] have been used to investigate aging in MDD, the novel DamAge and AdaptAge clocks have not yet been explored in this context.

Furthermore, identifying targetable biomolecules that modify DNA methylation age acceleration would help to understand the underlying biological aging processes, which could further provide biomarkers for interventions. Previous genetic studies showed epigenetic clocks are influenced by genetic factors and have heritability estimates ranging from 0.1 to 0.43 [34, 35]. Genome-wide association studies (GWAS) have been used to elucidate the genetic architecture of epigenetic aging. For example, Gibson et al. identified 10 independent SNPs associated with Horvath-based epigenetic age acceleration, implicating pathways in metabolism and immune regulation [36]. McCartney et al. expanded this landscape through a meta-analysis of 40,905 individuals, discovering 137 genetic loci linked to four epigenetic clocks and identifying shared genetic loci associated with the Horvath clock and expression of transcripts encoding genes involved in lipid metabolism and immune function [35]. Mavromatis et al. further employed multi-omics analysis and identified several important biological processes, cellular components and molecular functions, including insulin response, negative regulation of gene expression [37]. Moreover, although genetic factors influencing epigenetic aging have been explored, the specific epigenetic markers driving age acceleration in MDD remain underexplored. Importantly, there is growing interest in the potential to modify epigenetic aging through therapeutic interventions. Over the past few decades, many studies have shown the therapeutic potential of natural products in epigenetic aging [38]. Moreover, recent clinical trials have explored pharmacological and lifestyle strategies to decelerate epigenetic aging [39, 40], highlighting the translational potential of targeting DNA methylation changes. These findings suggest that future research should explore the potential for targeted interventions to mitigate epigenetic aging in depressed patients, which may ultimately provide novel opportunities for the prevention and treatment of aging-related comorbidities in MDD.

In addition, individuals with MDD frequently exhibit cognitive impairments in areas such as learning and memory, executive function, processing speed, and attention. Furthermore, these cognitive dysfunctions are considered an endophenotype of MDD that deteriorates with the frequency of depressive episodes [41-43]. Converging evidence indicates that age affects the type and severity of cognitive dysfunction in MDD [44, 45]. Elderly individuals with MDD frequently display more pronounced neurocognitive deficits compared to younger groups with MDD [46]. In recent years, an increasing number of studies have investigated the relationship between epigenetic aging processes and cognitive abilities. These studies consistently find that accelerated epigenetic aging is associated with impaired cognitive performance [47-54]. The Cambridge Neuro-psychological Testing Automated Battery [55] (CANTAB) is a neuropsychological measure developed at the University of Cambridge. CANTAB tests are noted for their sensitivity in detecting changes in cognitive functioning. Its correlation with epigenetic aging in MDD patients has not been examined.

In this study, we aim to explore the following key questions: (1) How are various epigenetic aging indicators, including the novel DamAge and AdaptAge clocks, associated with MDD? (2) What are the underlying molecular mechanisms linking epigenetic aging and MDD, as revealed by epigenome-wide association studies (EWAS)? (3) How does epigenetic aging relate to specific cognitive functions in MDD patients. We comprehensively examined the association between epigenetic aging and MDD using 13 widely used epigenetic clocks. We then investigated the molecular pathways underlying depression-related aging by conducting an EWAS with the most significant epigenetic aging indicator. Additionally, we applied selected tests from CANTAB to explore the relationship between specific cognitive functions and epigenetic aging in patients with MDD.

## MATERIALS AND METHODS

### Participants

This study used two datasets for epigenetic clock calculation. The discovery dataset included 39 patients with MDD and 40 healthy controls. The patients were recruited from the outpatient psychiatric department of Peking University Sixth Hospital, with diagnoses established by clinical psychiatrists based on the criteria outlined in the Diagnostic and Statistical Manual of Mental Disorders, Fifth Edition (DSM-5). Exclusion criteria for patients included a history of comorbid major psychiatric disorders, primary neurological diseases. All patients were assessed using the 24-item Hamilton Depression Rating Scale [56] (HAMD) and the Beck Depression Inventory [57] (BDI). The healthy controls, matched for age and gender, were recruited from the community, with exclusion criteria similar to those of the patient group. All healthy controls were assessed using the BDI.

The replication dataset comprising 359 MDD cases compared to 68 healthy controls was downloaded from GSE198904 [58]. MDD patients were obtained from the Molecular Biomarkers of Antidepressant Response study [59, 60]. Patients were required to have a current major depressive episode diagnosis according to the SCID-I and a HAMD-21 score of ≥20. Additionally, 68 control samples were recruited through advertisement. Among the participants, 66.2% of healthy controls and 61.0% of MDD cases were female. Additionally, 79.7% of the MDD cases and 79.4% of the healthy controls were of European ancestry. More details can be found in the original publications.

The study protocol was reviewed and approved by the Medical Ethics Committee of Peking University Sixth Hospital. All participants provided written informed consent prior to participation. The research was conducted in accordance with the Declaration of Helsinki and complied with all relevant ethical guidelines and regulations.

### Cognitive assessments

Cognitive assessments of patients with MDD in the discovery dataset were conducted using the CANTAB. The specific assessments included: (1) Cambridge Gambling Task (CGT): this task evaluates decision-making and risk-taking behavior; (2) Delayed Matching to Sample (DMS): this task assesses both simultaneous visual matching ability and short-term visual recognition memory; (3) Emotional Bias Task (EBT): this task detects perceptual biases in facial emotion perception; (4) Emotion Recognition Task (ERT): this task measures the ability to identify six basic emotions in facial expressions along a continuum of expression magnitude; (5) One Touch Stockings of Cambridge (OTS): this task evaluates executive functions, specifically spatial planning and working memory; (6) Rapid Visual Information Processing (RVP): this task measures sustained attention and psychomotor speed; (7) Spatial Working Memory (SWM): this task requires participants to retain and manipulate visuospatial information, providing insights into their working memory capabilities. We selected several key metrics from these tasks for further analysis (Supplementary Table 1).

### DNA methylation measurement

Genomic DNA was extracted from whole blood samples, and genome-wide DNA methylation profiling was conducted using the Infinium MethylationEPIC v2.0 BeadChip for the discovery dataset and the Infinium MethylationEPIC BeadChip for the replication dataset. The same preprocessing of DNA methylation data was performed for both datasets. The raw IDAT files were processed with the R package ChAMP [61]. For quality control during data loading, the following criteria were applied: (1) probes with a detection P-value greater than 0.01, indicating relatively low reliability; (2) probes that had fewer than 3 beads in at least 5% of the samples; (3) probes that were non-CpG, SNP related, or multi-hit probes, which could lead to technical artifacts and affect the reliability of methylation measurements; (4) probes located on the X or Y chromosomes. After applying quality control measures, 873,847 CpG probes of discovery dataset were retained for subsequent analysis. The Beta Mixture Quantile Dilation (BMIQ) method was utilized to normalize the beta values. Additionally, we employed the ComBat algorithm to correct for batch effects, which uses an empirical Bayes framework to adjust for technical variations.

### Epigenetic age calculation

For both discovery and validation datasets, 13 epigenetic clocks from the Biolearn [62] python package (Table 1) were calculated. We included 13 widely used epigenetic clocks to not only provide a comprehensive assessment of epigenetic aging in MDD without bias toward any single clock, but also presented their correlations with each other. Among them, HorvathV1 [21], HorvathV2 [63], Hannum [22], Lin [64], PhenoAge [23], CausAge [26], DamAge [26], AdaptAge [26], GrimAgeV1 [24], and GrimAgeV2 [65] were used to predict epigenetic age. Age acceleration (AgeAccel) was defined as the residual from the regression of epigenetic age on chronological age. Notably, DamAge correlates with adverse outcomes, including mortality, whereas AdaptAge is associated with beneficial adaptations. The Zhang [25] clock was applied to predict mortality risk, while DunedinPoAm [66] and DunedinPACE [67] were designed to measure the pace of biological aging (Aging Rate: Years/Year).

**Table 1:** Descriptions of the 13 epigenetic clocks.

Clock	Description	AgeAccel	MDD (Mean ± SD)	Control (Mean ± SD)	P vaule
**Predict epigenetic age**					
HorvathV1	353 CpG sites; multi-tissue predictor of age.	AgeAccelHor1	0.541 ± 5.284	-0.528 ± 3.998	0.3150
HorvathV2	391 CpG sites; DNAm age estimator for fibroblasts and other cell types used in ex vivo studies.	AgeAccelHor2	0.463 ± 4.73	-0.451 ± 2.109	0.2744
Hannum	71 CpG sites; predictor of age.	AgeAccelHan	0.287 ± 3.772	-0.28 ± 2.335	0.4261
Lin	99 CpG sites; predictor of age.	AgeAccelLin	0.694 ± 6.522	-0.677 ± 4.673	0.2877
PhenoAge	513 CpG sites; incorporating clinical phenotypic age measures that reflect variations in lifespan and healthspan to develop an age predictor.	AgeAccelPhe	0.503 ± 5.463	-0.49 ± 4.274	0.3719
CausAge	586 CpG sites; causality-enriched epigenetic clock.	AgeAccelCau	0.524 ± 5.064	-0.511 ± 2.909	0.2714
DamAge	1090 CpG sites; the damaging clock, which contains only the damaging CpG sites.	AgeAccelDam	2.357 ± 7.079	-2.298 ± 5.385	0.0016
AdaptAge	1000 CpG sites; the protective clock, which contains only the adaptive CpG sites.	AgeAccelAda	-1.138 ± 5.512	1.109 ± 5.619	0.0767
GrimAgeV1	1125 CpG sites; a composite epigenetic clock based on DNA methylation surrogates of plasma proteins and smoking history.	AgeAccelGrim1	0.323 ± 2.29	-0.315 ± 1.61	0.1571
GrimAgeV2	1346 CpG sites; a composite epigenetic clock combining DNA methylation surrogates of two new plasma proteins, high sensitivity CRP and hemoglobin A1C.	AgeAccelGrim2	0.446 ± 2.636	-0.435 ± 1.744	0.0850
**Predict pace of aging**					
DunedinPoAm	46 CpG sites; measurements of the pace of aging	-	0.929 ± 0.021	0.92 ± 0.019	0.0486
DunedinPACE	173 CpG sites; incorporating a longer follow-up period to measure the pace of aging	-	1.01 ± 0.072	0.99 ± 0.061	0.1873
**Predict mortality risk**					
Zhang	10 CpG sites; predictor of mortality risk.	-	-2.735 ± 0.268	-2.862 ± 0.198	0.0192

Note: AgeAccel stands for age acceleration. The Mean ± SD values for both MDD and control groups were calculated based on three categories of epigenetic measures: 10 epigenetic clocks that predict epigenetic age, two pace-of-aging measures, and one mortality risk estimator. P-values were obtained using two-sample t-tests to evaluate differences between the case (n = 39) and control (n = 40) groups.

### Statistical analysis

For the analysis of the 13 epigenetic clocks, we employed a two-sample t-test to evaluate differences between the case and control groups. Subsequently, linear regression models were used to examine the association between the epigenetic clocks and BDI scores, adjusting for potential confounders, including sex, smoking, drinking, and BMI. The analysis for the association of epigenetic clocks with cognition measures was also based on a similar linear regression model.

### Epigenome-wide association study of age acceleration of DamAge clock

To identify CpG sites associated with age acceleration of the DamAge clock, mixed linear models were implemented using the “DML” function from the SeSAMe [68] package in R, adjusting for sex, smoking, drinking, BMI, and batch effects. Significant CpG sites were determined after Bonferroni correction (0.05/873,847). The identified CpG sites were mapped to CpG islands and genes using the Annotatr [69] package in R, and pathway enrichment analysis was conducted using Metascape [70].

## RESULTS

### Demography of participants

The discovery dataset included 39 patients with MDD and 40 healthy controls. There were no significant differences between the MDD and control groups in terms of chronological age, sex distribution, BMI, or smoking and drinking. The BDI scores were significantly higher in the MDD group (31.46 ± 9.63) compared to the control group (3.08 ± 2.96), with a P-value of < 2.2e-16. Additional characteristics of the participants are summarized in Table 2.

### Elevated epigenetic aging and mortality risk in MDD

**Figure 1.**
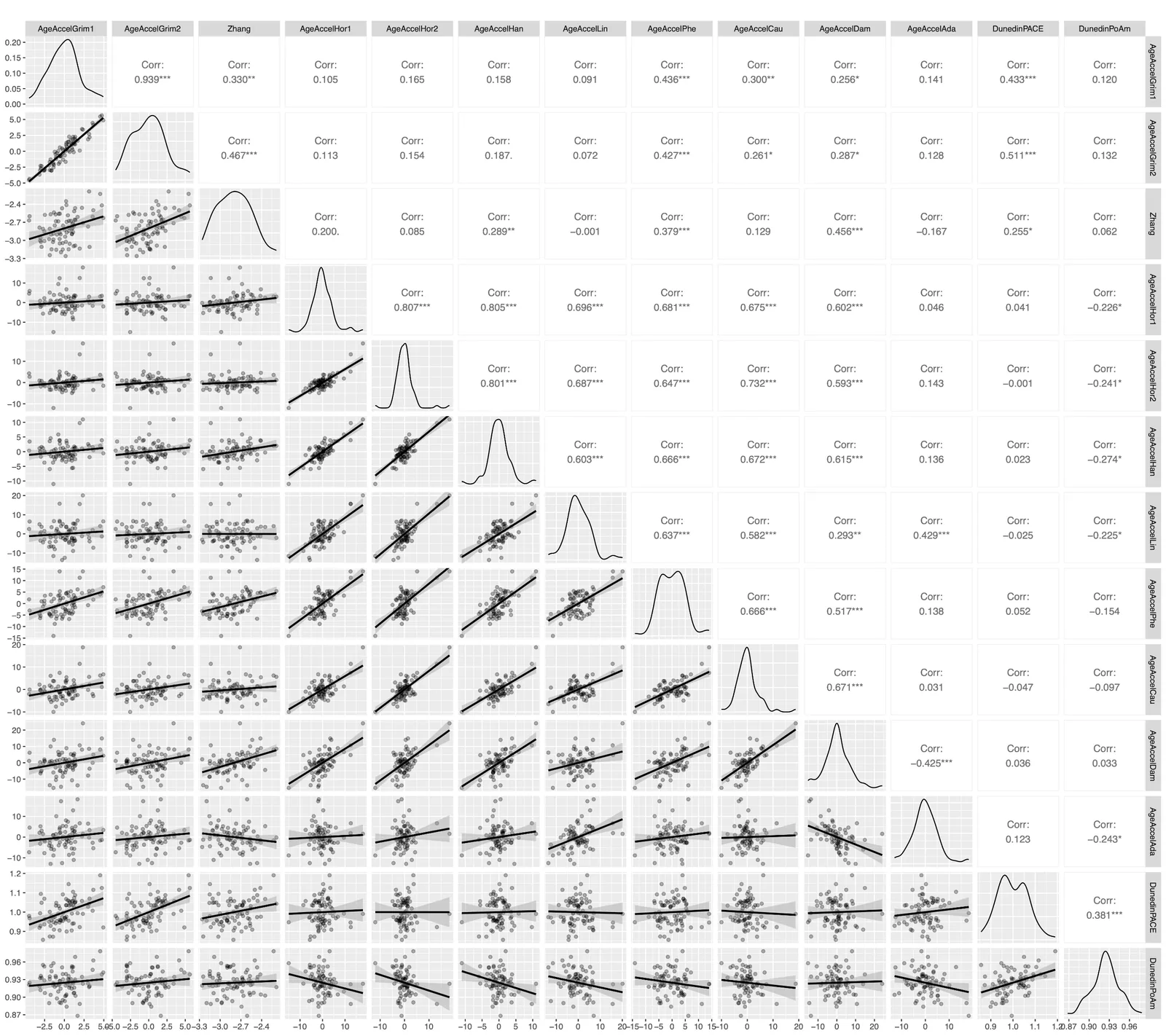
**Pairwise correlations among 13 clocks in the discovery dataset**. The plots on the diagonal show the distribution of each clock. The lower left triangular panels display scatter plots between pairs of clocks. The upper right triangular panels present Pearson correlation coefficients. Statistical significance of the correlations is indicated by asterisks: ^*^ denotes P < 0.05, ^**^ denotes P < 0.01, and ^***^ denotes P < 0.001.

**Figure 2.**
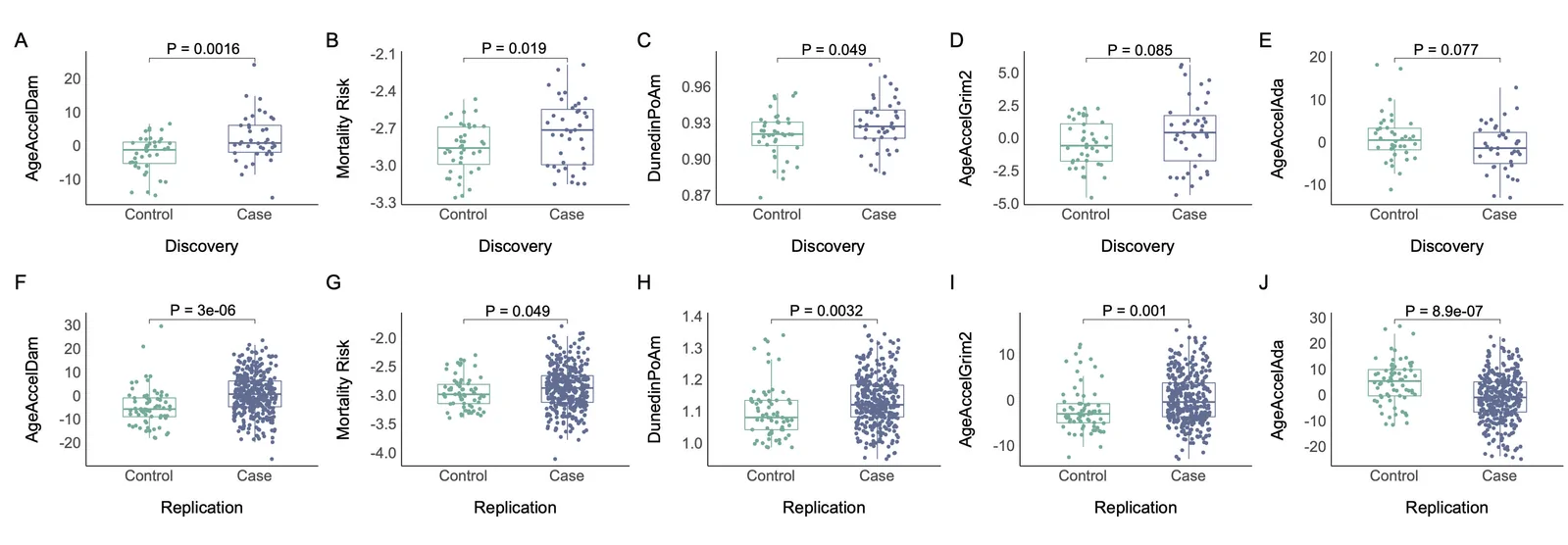
**Age acceleration and mortality risk comparisons between patients with MDD and healthy controls**. (**A**) Age acceleration from the DamAge clock. (**B**) Mortality risk estimated by the Zhang clock. (**C**) Aging rate measured by the DunedinPoAm clock. (**D**) Age acceleration from the GrimAgeV2 clock. (**E**) Age acceleration from the AdaptAge clock. (**F-J**) Validation of findings in an independent dataset. P-values were obtained using two-sample t-tests to evaluate differences between the case (n = 39) and control (n = 40) groups.

We calculated 13 epigenetic clocks, including 10 clocks for age acceleration, one clock for mortality risk estimated by the Zhang clock, and two clocks for aging rates measured by the DunedinPoAm and DunedinPACE clocks based on the DNA methylation data. The correlations among these 13 clock measures in the discovery and validation sets indicate that, most of the indicators show positive correlations with each other (Fig. 1, Supplementary Fig. 1), however, DamAge (AgeAccelDam) and AdaptAge (AgeAccelAda) showed strong negative correlation. Then, we performed group comparisons between patients with MDD and healthy controls for these 13 clocks. The analysis revealed that AgeAccelDam was significantly higher in MDD patients compared to healthy controls (t = 2.004, P = 0.0016, Cohen’s d = 0.451, Fig. 2A) in discovery dataset. Furthermore, the mortality risk estimated by the Zhang clock was significantly elevated in MDD patients relative to controls (t = 2.397, P = 0.019, Cohen’s d = 0.541, Fig. 2B). Moreover, the aging rate measured by the DunedinPoAm clock was also significantly higher in MDD patients than in healthy controls (t = 3.283, P = 0.049, Cohen’s d = 0.742, Fig. 2C).

**Table 2:** Participant demographics.

Variable	N (MDD/Control)	MDD	Control	Test
Age (years, Mean ± SD)	39/40	24.46 ± 7.48	22.68 ± 3.70	t = 1.34, P = 0.19
BMI (Mean ± SD)	39/40	21.76 ± 3.97	21.17 ± 2.59	t = 0.78, P = 0.44
BDI (Mean ± SD)	37/40	31.46 ± 9.63	3.08 ± 2.96	t = 17.19, P < 2.2e-16
HAMD (Mean ± SD)	35/0	25.89 ± 7.82	-	-

Sex	39/40	-	-	chi-square = 0.33, P = 0.56
Male (N)	-	12	9	
Female (N)	-	27	31	

Smoking	39/40	-	-	chi-square = 2.45, P = 0.12
Yes	-	4	0	
No	-	35	40	

Drinking	39/40	-	-	chi-square = 3.53, P = 0.06
Yes	-	5	0	
No	-	34	40	

Interestingly, we also observed that the age acceleration of the GrimAgeV2 clock (AgeAccelGrim2) was higher in patients with MDD compared to healthy controls, although this difference was only on marginal significance (P = 0.085, Fig. 2D). Notably, the GrimAge clock is a clock that shows a significant association with mortality risk, and the age acceleration of the GrimAgeV1 clock has been shown to be significantly higher in patients with MDD compared to healthy controls [33]. Additionally, the age acceleration of AdaptAge (AgeAccelAda), which is associated with beneficial adaptations, was lower in the patient group, although the difference did not reach significance (P = 0.077, Fig. 2E). Moreover, using linear regression analysis adjusting for sex, smoking, drinking, and BMI, we found that AgeAccelDam (P = 0.005) and mortality risk (P = 0.02) remained significant.

We validated our findings using validation dataset and found that AgeAccelDam, mortality risk, DunedinPoAm, and AgeAccelGrim2 were significantly higher in the MDD group compared to the healthy controls (Fig. 2F-I). Additionally, AgeAccelAda was significantly lower in the MDD group (Fig. 2J).

### AgeAccelDam correlates with depressive symptoms: EWAS and functional annotation

**Figure 3.**
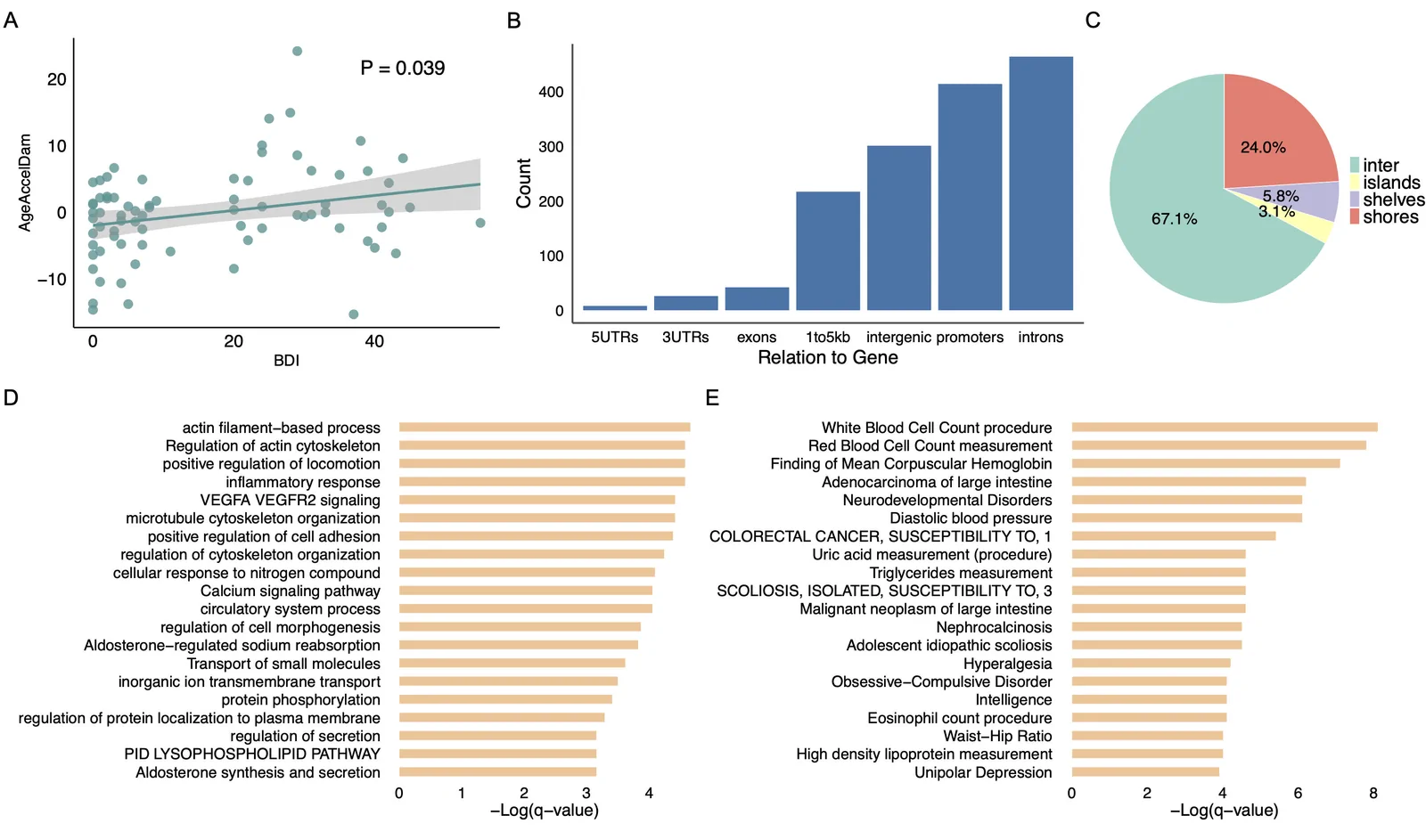
**Associations between BDI with AgeAccelDam and EWAS results**. (**A**) Correlation between AgeAccelDam and BDI scores. P-value were obtained from linear regression analyses adjusting for sex, smoking, drinking, and BMI. (**B**) Distribution of significant CpG sites across gene regions. (**C**) Annotation of significant CpG sites relative to CpG Island features. (**D**) Top 20 gene set clusters with their representative enriched gene set terms (one per cluster) from Metascape. q-values are calculated using the Benjamini-Hochberg procedure to account for multiple testing. (**E**) Top 20 enriched diseases identified by DisGeNET.

To investigate the association between AgeAccelDam and mortality risk with depressive symptoms, we performed linear regression analyses adjusting for sex, smoking, drinking, and BMI. Our results revealed a significant positive correlation between AgeAccelDam and BDI scores (P = 0.039, Fig. 3A). Additionally, we also explored the relationships of AgeAccelDam and mortality risk with HAMD scores within the MDD group but did not find significant results.

We performed EWAS to investigate the molecular mechanisms underlying age acceleration measured by the DamAge clock. After applying Bonferroni correction for multiple testing, 1,472 CpG sites were identified as significantly associated with AgeAccelDam (Supplementary file 1). Notably, all of these CpG sites showed negative estimates, with effect sizes ranging from -0.0008 to -0.0059. In the validation cohort, 960 of these 1,472 CpG sites were available for analysis. Among them, 957 of these CpG sites were successfully validated (P < 0.05/960, Supplementary file 2), all showing negative effect directions consistent with the discovery cohort. Intriguingly, only four of the 1,472 CpGs corresponded to known markers of the DamAge clock, suggesting that the majority of associations extend beyond the markers of DamAge clock (Supplementary file 3) and may reflect novel epigenetic changes related to age acceleration in MDD pathophysiology.

Annotation of the 1,472 significant CpG sites revealed that the majority were located within gene regulatory regions, including promoters (28.1%), 1-5 kb upstream regions (14.7%), and introns (31.5%) (Fig. 3B). Introns are recognized as important regulatory elements that can enhance gene expression through multiple mechanisms such as intron-mediated enhancement (IME), affecting transcription, and the efficiency of mRNA translation [71, 72]. This distribution indicates that epigenetic alterations associated with AgeAccelDam in MDD are predominantly enriched in genomic regions with regulatory potential, suggesting their involvement in modulating gene expression. Additionally, annotation of the significant CpG sites relative to CpG island features showed that most of them are not located within CpG islands but are distributed predominantly in open sea regions (Fig. 3C).

To elucidate the biological processes and pathways, we performed enrichment analysis using Metascape [70] on the genes annotated from the 1,472 significant CpGs (Supplementary file 4, Fig. 3D). The results revealed significant enrichment in biological processes primarily related to cytoskeletal mechanisms, as well as signaling pathways including calcium signaling, small molecule transport, and protein phosphorylation. Additionally, enrichment of inflammatory response and cellular response to nitrogen compounds suggests an involvement of immune-related functions in the epigenetic aging of MDD.

Further enrichment analysis based on the DisGeNET [73] database showed that these genes are significantly associated with multiple clinical phenotypes and diseases, including unipolar depression, neurodevelopmental disorders, obsessive-compulsive disorder, adenocarcinoma of the large intestine, and susceptibility to colorectal cancer, among other neuropsychiatric and somatic conditions (Supplementary file 5, Fig. 3E). Moreover, traits related to blood cell counts (such as white and red blood cell counts), lipid profiles, and blood pressure were also enriched, indicating that epigenetic aging linked to depression may involve cross-system mechanisms encompassing immune regulation, metabolism, and cardiovascular function. These findings suggest that epigenetic aging in depression not only reflect the biological basis of neuropsychiatric disorders but are also connected to shared pathological processes underlying certain physical diseases.

### The relationship between cognitive functions and epigenetic aging

**Figure 4.**
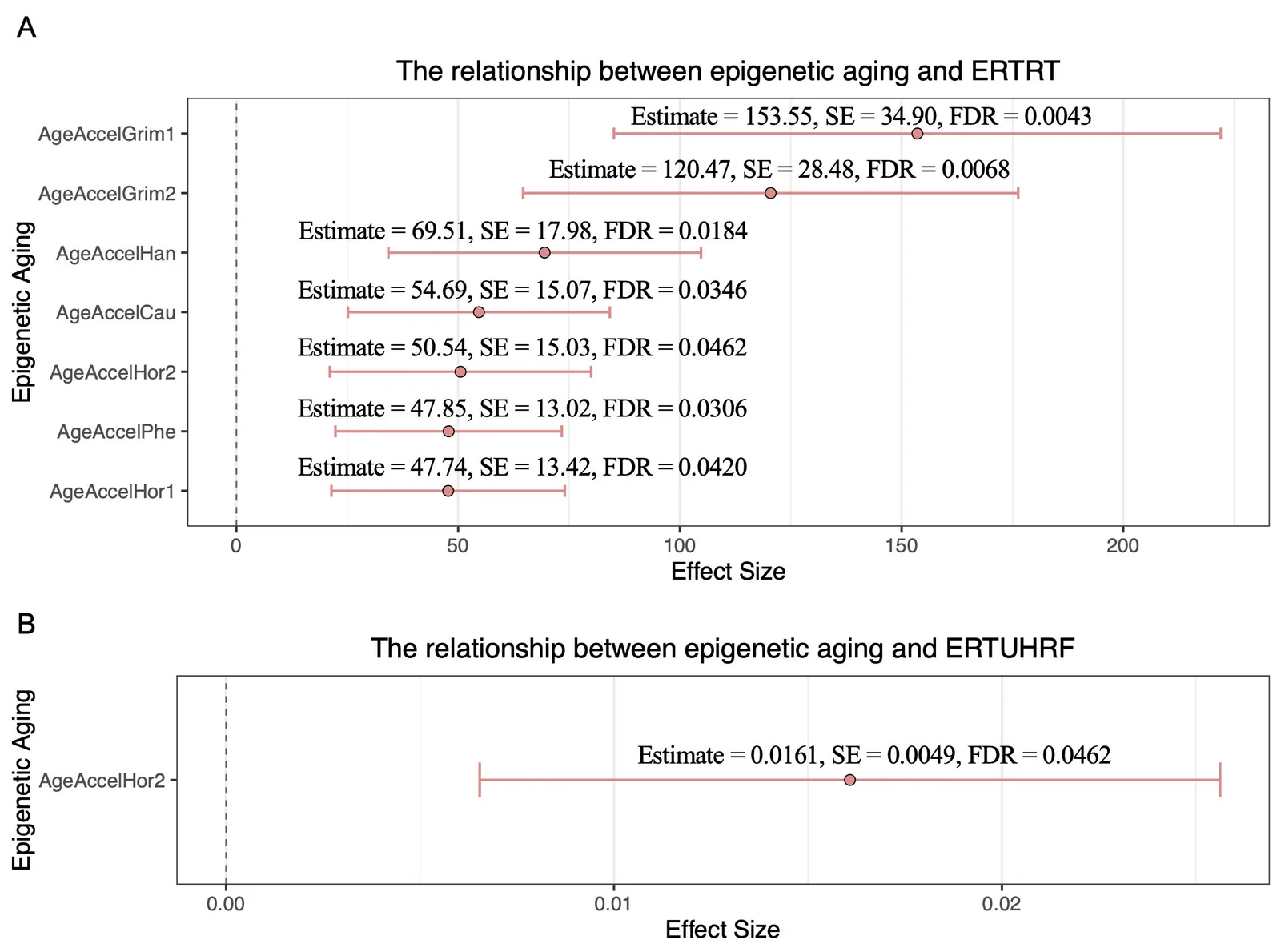
**Significant relationship between cognitive functions and epigenetic aging in patients with MDD**. (**A**) Correlation between Emotion Recognition Task Median Reaction Time (ERTRT) and age acceleration from seven epigenetic clocks. (**B**) Correlation between Emotion Recognition Task Unbiased Hit Rate for Fear (ERTUHRF) and age acceleration from HorvathV2 clock. Linear regression analyses adjusting for sex, smoking, drinking, and BMI were performed to investigate the relationship between epigenetic aging and cognitive function. The significance levels shown in the figure are false discovery rate (FDR)-adjusted values.

We conducted linear regression analyses to investigate the relationship between cognitive function and epigenetic aging. The results indicated significant positive correlations between Emotion Recognition Task Median Reaction Time (ERTRT) and age acceleration calculated from seven epigenetic clocks (False discovery rate (FDR) < 0.05, Fig. 4A). Additionally, AgeAccelDam was positively correlated with ERTRT (Effect size = 30.68, SE = 10.60, P = 0.007), although it did not reach statistical significance after FDR correction. Furthermore, the Emotion Recognition Task Unbiased Hit Rate for Fear (ERTUHRF), which measures recognition accuracy for the fear emotion without being affected by guessing or response bias, demonstrated significant positive correlations with the age acceleration of HorvathV2 (AgeAccelHor2) (FDR < 0.05, Fig. 4B). Other CANTAB domains did not reach significance after FDR correction, although several showed nominal significances (Supplementary file 6).

## DISCUSSION

In this study, we comprehensively compared the correlation of 13 epigenetic clocks and explored the relationship between epigenetic aging and MDD. Our findings revealed that AgeAccelDam was significantly higher in patients with MDD compared to healthy controls, as well as the mortality risk measured by the Zhang clock. We further found significant positive correlations between AgeAccelDam and BDI scores. Additionally, our EWAS of AgeAccelDam identified 1,472 significant CpG sites predominantly enriched in gene regulatory regions. Furthermore, we investigated the relationship between cognitive function and epigenetic aging. Our results indicated significant positive correlations between emotion recognition deficits and age acceleration.

Individuals with a history of depression in youth faced significantly higher relative and absolute risks of being diagnosed with various medical conditions and experiencing early mortality compared to the general population [74]. The relationships between epigenetic aging and factors like morbidity and mortality suggest that epigenetic clocks could be useful tools for predicting and identifying risks related to aging [75]. Previous research has demonstrated epigenetic aging in MDD through the use of the GrimAge metric, which shows a strong correlation with morbidity and mortality [33]. In our study, both the age acceleration measured by GrimAgeV2, DamAge, and DunedinPoAm, as well as the mortality risk assessed by the Zhang clock, were found to be higher in patients. Previous studies have also found that an increased burden of psychiatric disorders, depression, and environmental factors such as incarceration exposure and childhood poverty are associated with age acceleration, as measured by the GrimAge and DunedinPoAm clocks [76-78]. Additionally, we found that the age acceleration measured by AdaptAge was lower in the MDD group. While this result was not statistically significant in the discovery set—likely due to limited sample size—it became significant in validation. Since AdaptAge reflects protective epigenetic changes against aging, a lower score in MDD may indicate reduced accumulation of such protective changes, suggesting impaired resilience in these individuals. The two clocks, DamAge and AdaptAge, have not been previously applied in studies of MDD. They underscore the importance of distinguishing between adaptive and damaging epigenetic changes in aging research, providing new insights into the mechanisms of aging.

This study revealed a positive correlation between depressive symptoms and epigenetic aging. The finding aligns with existing literature, which indicates that depressive symptoms are linked to higher levels of DNA methylation age acceleration [28, 79]. Our study is the first to demonstrate that AgeAccelDam is associated with depressive symptoms, highlighting the potential of biological aging as a key health indicator in depressed individuals. These insights underscore the importance of considering biological aging in the clinical assessment and treatment of depression, suggesting that monitoring and intervening in epigenetic aging could enhance patient outcomes.

Since AgeAccelDam is significantly associated with MDD, exploring the underlying biological mechanisms may help uncover the pathophysiology of MDD. Functional enrichment analysis revealed that the genes annotated from AgeAccelDam-related CpGs were primarily involved in cytoskeletal, signaling, and immune pathways. Moreover, DisGeNET enrichment analysis indicated that epigenetic aging may involve cross-system mechanisms encompassing immune regulation, metabolism, and cardiovascular function. These findings are consistent with broader evidence from independent studies. For instance, GWAS have demonstrated that metabolic and immune pathways share common genetic underpinnings with epigenetic clocks [36]. Consistently, GO enrichment analysis of genes from transcriptional analysis with the age acceleration of PhenoAge revealed significant enrichment in pro-inflammatory signaling pathways and antiviral response pathways [23]. Furthermore, the significant CpG sites identified in EWAS exhibit relatively small effect sizes and all show negative associations with AgeAccelDam. This pattern suggests that individual CpG sites may not act independently, but rather function collectively through shared biological pathways to influence epigenetic aging. Targeting these pathways could provide novel therapeutic opportunities to modulate epigenetic aging.

One question we addressed was whether the AgeAccelDam-related CpGs overlap with the 1,090 CpG sites used to calculate the DamAge clock. Comparison revealed only 4 overlapping CpGs between the two sets, indicating largely distinct marker compositions. Further analysis of their genomic distributions showed notable differences (Supplementary Fig. 2). The DamAge clock markers displayed a relatively balanced distribution, with a substantial proportion located within CpG islands and shore regions. In contrast, most AgeAccelDam-associated CpGs were situated in open sea regions. Functional enrichment analysis of the original DamAge clock CpG markers revealed predominant involvement in fundamental developmental processes and cell morphogenesis, consistent with the notion that the DamAge clock robustly captures the effects of age-related biological conditions. In contrast, the EWAS of AgeAccelDam in the context of MDD, along with its corresponding enrichment results, aimed to identify CpG sites and biological pathways potentially influencing damage-related biological age acceleration specifically in MDD. These AgeAccelDam-associated CpGs were enriched in pathways related to cytoskeletal organization, calcium signaling, small molecule transport, protein phosphorylation, as well as immune functions such as inflammatory response and cellular response to nitrogen compounds. This divergence suggests that the age acceleration of DamAge observed in MDD may be driven by additional, disease-specific molecular processes, particularly involving immune and signaling pathways that could contribute to the pathophysiology of accelerated biological aging in depression.

Participants with MDD often experience interpersonal functioning impairments. One significant contributing factor to these difficulties may be the impairment in recognizing facial expressions of emotion [80-82]. A meta-analysis suggests that the emotion recognition deficits on depression exist across all basic emotions except sadness [82]. However, the underlying mechanisms remain unclear. In our study, we found that epigenetic aging, as measured by seven different epigenetic clocks, was positively correlated with reaction time for emotion recognition in depressed patients. Although the association for AgeAccelDam did not survive FDR correction, it showed nominal significance. These results suggest that accelerated biological aging may be associated with slower emotional recognition responses. Further studies with larger sample sizes are needed to validate these findings. Additionally, we also observed that recognition accuracy for fear emotions correlated positively with epigenetic aging. It has been reported that individuals with depression exhibit increased vigilance and selective attention toward negative emotions [80]. Furthermore, another study found that MDD patients with suicidal ideation demonstrated more accurate recognition of fear emotions compared to those without suicidal ideation [83]. These findings not only extend prior research on emotional processing impairments in depression but also suggest that accelerated epigenetic aging may contribute to altered mechanisms underlying emotion recognition deficits. This highlights a potential biological pathway linking epigenetic aging processes with the emotional processing observed in MDD. The lack of significance in the other cognition domains may suggest a true absence of association with epigenetic aging, but it is also possible that these non-significant findings were influenced by limitations in sample size and statistical power, which might have hindered the detection of more subtle effects.

Our research has certain limitations that should be acknowledged. Firstly, while our findings of epigenetic aging have been validated in a larger independent cohort, we acknowledge that the analyses related to the EWAS and cognitive function were conducted only on a relatively small sample size. Despite the limited sample size, these results represent novel and significant findings in the field, warranting further investigation in larger cohorts to deepen our understanding. Secondly, since this study employed a cross-sectional design with data collected at a single time point, our ability to infer causal relationships is inherently limited. In contrast, longitudinal studies, by following the same participants across multiple time points, enable researchers to observe the temporal sequence of changes in variables, thereby helping to clarify the order of alterations among epigenetic aging, depression, and emotion recognition abilities. Thirdly, although we adjusted for sex, smoking, drinking, BMI, and batch effects, other potential confounders such as medication use, illness duration, and comorbidities may also influence our results. However, as these factors are often closely linked to the disorder itself—for example, antidepressant use is highly confounded with MDD, and adjusting for it may remove biological signals related to the disorder rather than treatment effects—we did not include them in our main analyses. This should be taken into consideration when interpreting our findings.

Overall, our findings extend previous research on epigenetic aging in MDD by highlighting the distinct contributions of damaging epigenetic changes through the novel DamAge clock. This study is the first to demonstrate a significant association between AgeAccelDam and depressive symptoms, as well as to investigate the underlying biological mechanisms. Additionally, we report for the first time a link between epigenetic aging and emotion recognition deficits in depression. While further research is needed to establish causality and predictive value, epigenetic aging markers hold promise as potential biomarkers to better understand individual differences in cognitive symptoms. Moreover, emerging evidence indicates that pharmacological agents, lifestyle modifications, and natural compounds targeting epigenetic aging pathways may offer novel avenues to mitigate biological aging and potentially improve clinical outcomes in MDD. Nonetheless, before considering the use of epigenetic aging markers in clinical practice, longitudinal studies are essential to confirm their utility and to elucidate the temporal and causal relationships between epigenetic aging and depressive symptoms. Future longitudinal studies are warranted to further elucidate the molecular pathways driving depression-related aging and to evaluate targeted interventions aimed at decelerating epigenetic aging in this vulnerable population.

## Supplementary Materials

The Supplementary data can be found online at: www.aginganddisease.org/EN/10.14336/AD.2025.0781.



## Data Availability

Supplementary Files 1-6 are available for download at: https://github.com/Chao-Liufu/MDD_clock. The original DNA methylation dataset, generated using the Infinium MethylationEPIC v2.0 BeadChip, is available upon reasonable request from the corresponding author.
