# Supplementary material for: Epigenetic Clocks Identify Harmful Epigenetic Aging Linked to Depression Severity and Cognitive Deficits in Major Depressive Disorder
Source: Aging Dis. 2026 Aug 18;17(5):2753–66. doi: 10.14336/AD.2025.0781 (PMC13437127; doi:10.14336/AD.2025.0781)
Supplement: Supplementary file 1 [file AD-17-5-2753-s.pdf]

SUPPLEMENTARY DATA

Epigenetic Clocks Identify Harmful Epigenetic Aging Linked to Depression Severity and Cognitive Deficits in Major Depressive Disorder

Liufu Chao, Tao Pang, Haohao Zheng, Lei Yang, Li Yang, Xuebing Huang, Ying Qian, Suhua Chang

© 2025. Zhao L et al. Published online at http://www.aginganddisease.org/EN/10.14336/AD.2025.0781

SUPPLEMENTARY DATA

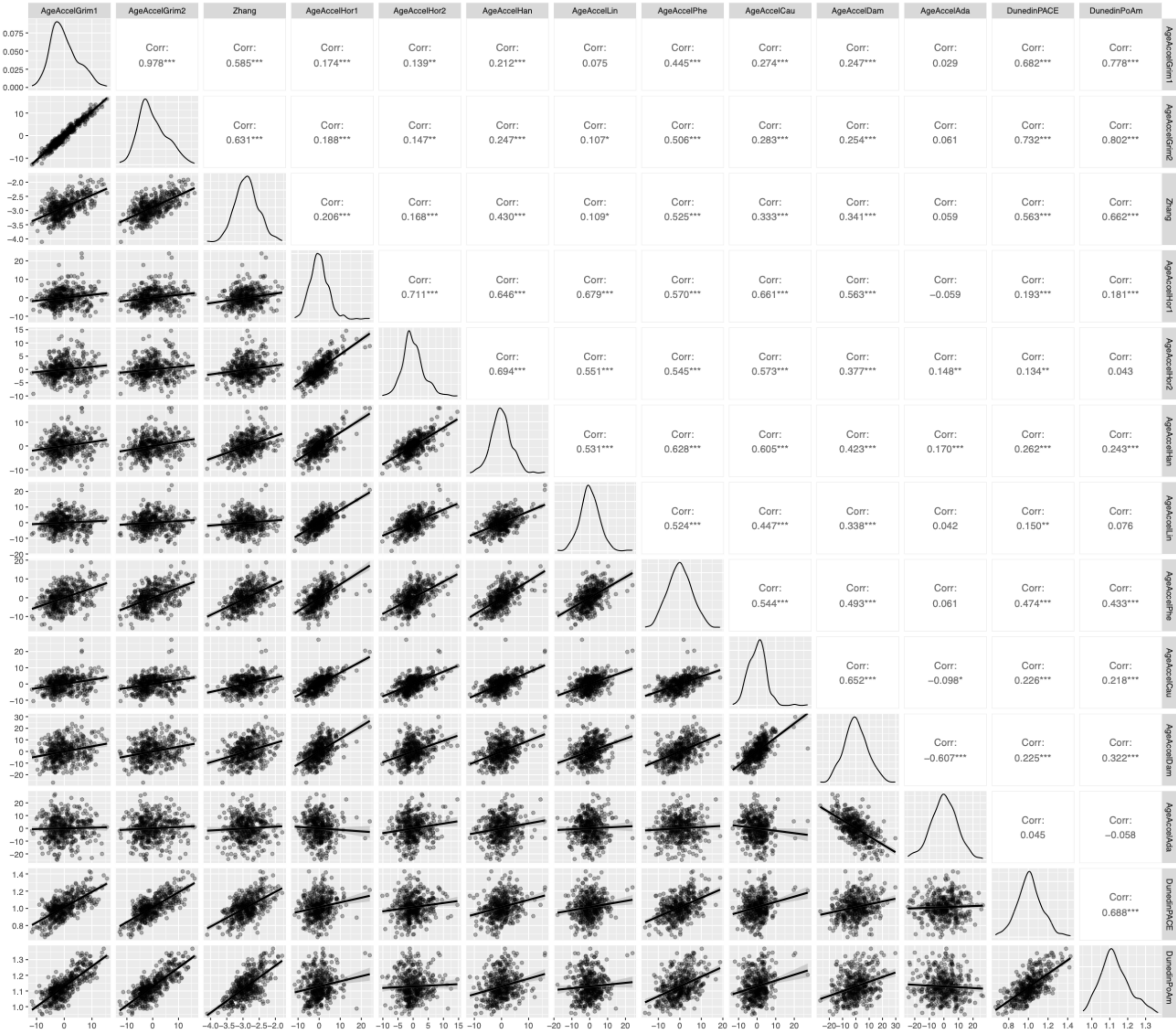

Supplementary Figure 1. Pairwise correlations among 13 clocks in the validation dataset. The plots on the diagonal show the distribution of each clock. The lower left triangular panels display scatter plots between pairs of clocks. The upper right triangular panels present Pearson correlation coefficients. Statistical significance of the correlations is indicated by asterisks: * denotes P < 0.05, ** denotes P < 0.01, and *** denotes P < 0.001.

© 2025. Zhao L et al. Published online at http://www.aginganddisease.org/EN/10.14336/AD.2025.0781

SUPPLEMENTARY DATA

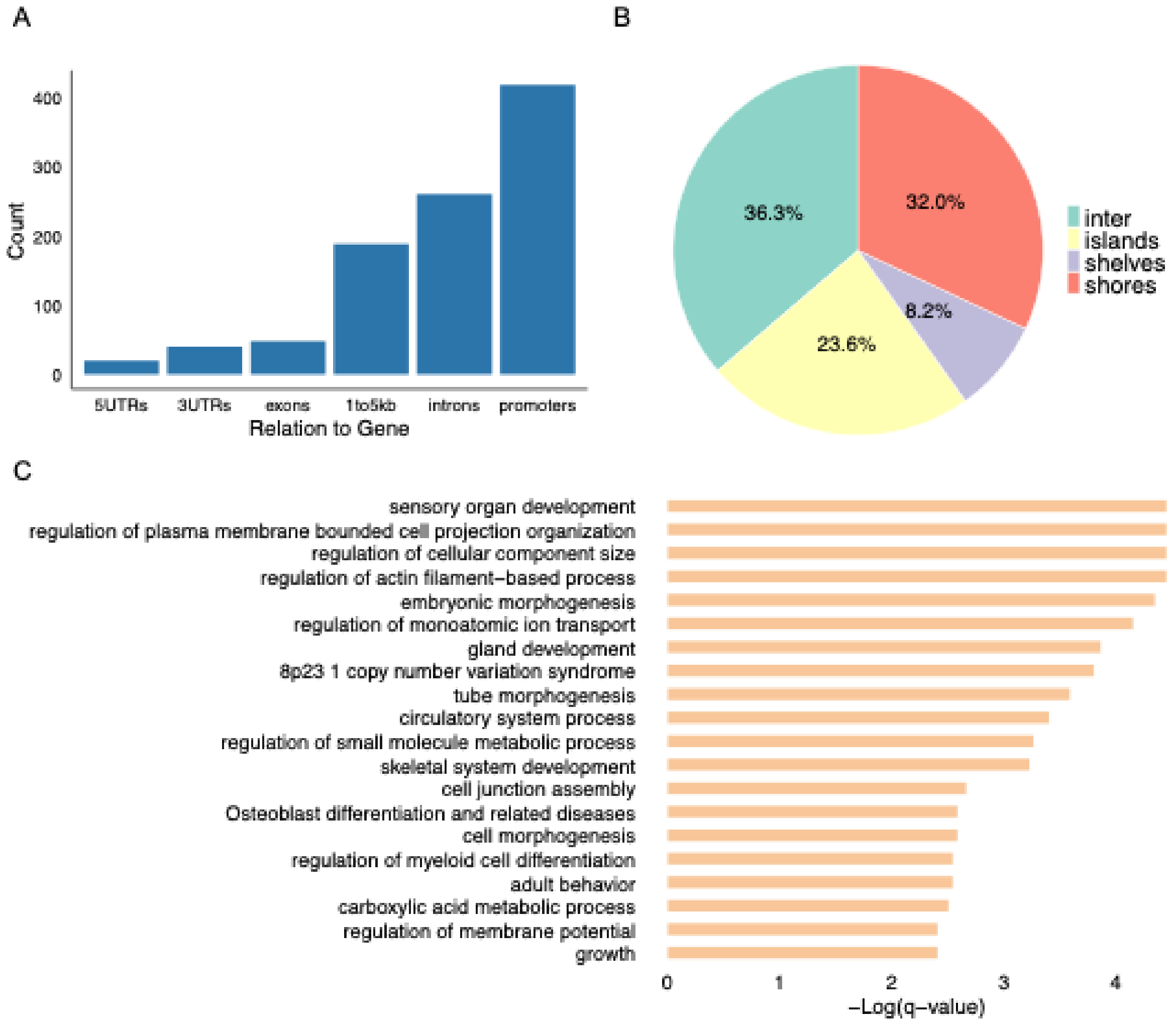

Supplementary Figure 2. The genomic distribution and functional features of DamAge clock markers. (A) Distribution of significant CpG sites across gene regions. (B) Annotation of significant CpG sites relative to CpG Island features. (C) Top 20 gene set clusters with their representative enriched gene set terms (one per cluster) from Metascape. q-values are calculated using the Benjamini-Hochberg procedure to account for multiple testing.

© 2025. Zhao L et al. Published online at http://www.aginganddisease.org/EN/10.14336/AD.2025.0781

SUPPLEMENTARY DATA

Supplementary Table 1. Cognitive assessments of CANTAB

Task	Cognition Function	Measure Name	Measure Description
CGT	Executive function	CGTDAVT	KEY: CGT Delay Aversion Total: Allows for the dissociation between risk taking and impulsivity by determining whether subjects simply just place a bet at the first opportunity. Calculated as CGT Risk Taking for all trials from the descending condition minus CGT Risk Taking for all trials from the ascending condition.
CGT	Executive function	CGTDMQMT	KEY: CGT Decision Making Quality Total Merged: The proportion (0 ? 1) of all trials where the subject chose the majority box colour. Calculated over all assessed trials from both the ascending and descending conditions in which the number of boxes of each colour differed.
CGT	Executive function	CGTOPBMT	CGT Overall Proportion Bet Total Merged: The mean proportion (0 - 1) of current points gambled by the subject. Calculated over all assessed trials from both the ascending and descending conditions.
CGT	Executive function	CGTRAJMT	KEY: CGT Risk Adjustment Merged: Risk adjustment is a measure of sensitivity to risk, based on the ability to modify choices in the light of information about the probability of different outcomes and to track the optimal outcome on each trial. The measure is calculated from the average proportion of points that the subject chose to bet with, taking into account the number of coloured boxes in the majority.
DMS	Memory	DMSPC0	KEY: DMS Percent Correct (0 seconds delay): The percentage of assessment trials containing a zero second delay during which the subject chose the correct box on their first box choice. Calculated across all assessed trials containing a zero second delay.
DMS	Memory	DMSPC12	KEY: DMS Percent Correct (12 second delay): The percentage of assessment trials containing a twelve second delay during which the subject chose the correct box on their first box choice. Calculated across all assessed trials containing a twelve second delay.
DMS	Memory	DMSPC4	KEY: DMS Percent Correct (4 second delay): The percentage of assessment trials containing a four second delay during which the subject chose the correct box on their first box choice. Calculated across all assessed trials containing a four second delay.
DMS	Memory	DMSPCAD	KEY: DMS Percent Correct (all delays): The percentage of assessment trials containing a delay during which the subject chose the correct box on their first box choice. Calculated across all assessed trials containing a delay.
DMS	Memory	DMSPCS	KEY: DMS Percent Correct (simultaneous): The percentage of assessment trials where the target and response stimuli were presented simultaneously during which the subject chose the correct box on their first box choice. Calculated across all assessed trials containing the simultaneous presentation of stimuli.
DMS	Memory	DMSPEGE	KEY: DMS Probability of Error Given Error: This measure reports the probability of an error occurring when the previous trial was responded to incorrectly. Calculated across all assessed trials (simultaneous and all delays).
EBT	Emotion & social cognition	EBTBP-HA	KEY: EBT Bias Point: The proportion of assessed trials where the subject selected 'Happy', adjusted to a scale of 0 to 15. (Number of assessed trials selected as 'Happy'/Number of all assessed trials)*15
EBT	Emotion & social cognition	EBTBP-HD	KEY: EBT Bias Point: The proportion of assessed trials where the subject selected 'Happy', adjusted to a scale of 0 to 15. (Number of assessed trials selected as 'Happy'/Number of all assessed trials)*15
EBT	Emotion & social cognition	EBTBP-HS	KEY: EBT Bias Point: The proportion of assessed trials where the subject selected 'Happy', adjusted to a scale of 0 to 15. (Number of assessed trials selected as 'Happy'/Number of all assessed trials)*15
ERT	Emotion & social cognition	ERTRT	KEY: ERT Median Reaction Time: The median latency for a subject to select an emotion after being presented with a stimulus. Calculated across all assessed trials.

© 2025. Zhao L et al. Published online at http://www.aginganddisease.org/EN/10.14336/AD.2025.0781

SUPPLEMENTARY DATA

ERT	Emotion & social cognition	ERTTH	KEY: ERT Total Hits: The total number of times the subject selected the correct emotion. Calculated across all assessed trials.
ERT	Emotion & social cognition	ERTUHRA	KEY: ERT Unbiased Hit Rate Anger: The unbiased hit rate ensures that recognition accuracy of the Anger emotion is not influenced by response guessing or response bias effects. It takes into consideration the joint probability of an individual making a correct response, based on the presentation of the correct stimulus out of the available possibilities. Calculated for assessed Anger trials only.
ERT	Emotion & social cognition	ERTUHRD	KEY: ERT Unbiased Hit Rate Disgust: The unbiased hit rate ensures that recognition accuracy of the Disgust emotion is not influenced by response guessing or response bias effects. It takes into consideration the joint probability of an individual making a correct response, based on the presentation of the correct stimulus out of the available possibilities. Calculated for assessed Disgust trials only.
ERT	Emotion & social cognition	ERTUHRF	KEY: ERT Unbiased Hit Rate Fear: The unbiased hit rate ensures that recognition accuracy of the Fear emotion is not influenced by response guessing or response bias effects. It takes into consideration the joint probability of an individual making a correct response, based on the presentation of the correct stimulus out of the available possibilities. Calculated for assessed Fear trials only.
ERT	Emotion & social cognition	ERTUHRH	KEY: ERT Unbiased Hit Rate Happiness: The unbiased hit rate ensures that recognition accuracy of the Happiness emotion is not influenced by response guessing or response bias effects. It takes into consideration the joint probability of an individual making a correct response, based on the presentation of the correct stimulus out of the available possibilities. Calculated for assessed Happiness trials only.
ERT	Emotion & social cognition	ERTUHRS	KEY: ERT Unbiased Hit Rate Sadness: The unbiased hit rate ensures that recognition accuracy of the Sadness emotion is not influenced by response guessing or response bias effects. It takes into consideration the joint probability of an individual making a correct response, based on the presentation of the correct stimulus out of the available possibilities. Calculated for assessed Sadness trials only.
ERT	Emotion & social cognition	ERTUHRSU	KEY: ERT Unbiased Hit Rate Surprise: The unbiased hit rate ensures that recognition accuracy of the Surprise emotion is not influenced by response guessing or response bias effects. It takes into consideration the joint probability of an individual making a correct response, based on the presentation of the correct stimulus out of the available possibilities. Calculated for assessed Surprise trials only.
OTS	Executive function	OTSMDLFC	KEY: OTS Median Latency to First Choice: The median latency, measured from the appearance of the stocking balls until the first box choice was made by the subject. Calculated across all assessed trials where the subject's first response was correct.
OTS	Executive function	OTSPSFC	KEY: OTS Problems Solved on First Choice: The total number of assessed trials where the subject chose the correct answer on their first attempt. Calculated across all assessed trials.
RVP	Attention & psychomotor speed	RVPA	KEY: RVP A?: A? (A prime) is the signal detection measure of a subject's sensitivity to the target sequence (string of three numbers), regardless of response tendency (the expected range is 0.00 to 1.00; bad to good). In essence, this metric is a measure of how good the subject is at detecting target sequences.
RVP	Attention & psychomotor speed	RVPMDL	KEY: RVP Median Response Latency: The median response latency on trials where the subject responded correctly. Calculated across all assessed trials.
RVP	Attention & psychomotor speed	RVPPFA	KEY: RVP Probability of False Alarm: The number of sequence presentations that were false alarms divided by the number of sequence presentations that were false alarms plus the number of sequence presentations that were correct rejections: (False Alarms ÷ (False Alarms + Correct Rejections))
SWM	Executive function	SWMBE12	KEY: SWM Between errors 12 boxes: The number of times the subject revisits a box in which a token has previously been found. Calculated across all trials with 12 tokens only.

© 2025. Zhao L et al. Published online at http://www.aginganddisease.org/EN/10.14336/AD.2025.0781

SUPPLEMENTARY DATA

SWM	Executive function	SWMBE4	KEY: SWM Between errors 4 boxes: The number of times a subject revisits a box in which a token has previously been found. Calculated across all trials with 4 tokens only.
SWM	Executive function	SWMBE468	KEY: SWM Between Errors: The number of times the subject incorrectly revisits a box in which a token has previously been found. Calculated across all assessed four, six and eight token trials.
SWM	Executive function	SWMBE6	KEY: SWM Between errors 6 boxes: The number of times the subject revisits a box in which a token has previously been found. Calculated across all trials with 6 tokens only.
SWM	Executive function	SWMBE8	KEY: SWM Between errors 8 boxes: The number of times the subject revisits a box in which a token has previously been found. Calculated across all trials with 8 tokens only.
SWM	Executive function	SWMS	KEY: SWM Strategy (6-8 boxes): The number of times a subject begins a new search pattern from the same box they started with previously. If they always begin a search from the same starting point, we infer that the subject is employing a planned strategy for finding the tokens. Therefore, a low score indicates high strategy use (1 = they always begin the search from the same box), a high score indicates that they are beginning their searches from many different boxes. Calculated across assessed trials with 6 tokens or 8 tokens.

© 2025. Zhao L et al. Published online at http://www.aginganddisease.org/EN/10.14336/AD.2025.0781
